# Preparedness for Future Pandemics: Lessons Learned From the COVID-19 Pandemic in Iran

**DOI:** 10.3389/ijph.2022.1605094

**Published:** 2022-06-29

**Authors:** Amirhossein Takian, Seyed Sahab Aarabi, Farbod Semnani, Amirmasoud Rayati Damavandi

**Affiliations:** ^1^ Department of Global Health and Public Policy, School of Public Health, Tehran University of Medical Sciences (TUMS), Tehran, Iran; ^2^ Department of Health Management, Policy and Economics, School of Public Health, Tehran University of Medical Sciences (TUMS), Tehran, Iran; ^3^ Health Equity Research Centre (HERC), Tehran University of Medical Sciences (TUMS), Tehran, Iran; ^4^ School of Public Health, Tehran University of Medical Sciences (TUMS), Tehran, Iran; ^5^ School of Medicine, Tehran University of Medical Sciences (TUMS), Tehran, Iran

**Keywords:** COVID-19, Iran, pandemic management, universal health coverage, intersectoral action

As we are becoming more resilient to the catastrophic burden of the COVID-19 pandemic, the threat of future outbreaks is still on the horizon. Since detecting the first COVID-19 case in Iran (19 February 2020), six pandemic waves have occurred, leading to more than 7 million confirmed cases and over 140 thousand official deaths [1]. As of 9 January 2021, The Lowy institute ranked Iran’s performance 95th out of 98 countries in battling the COVID-19 pandemic [2], emphasizing vigilance against unforeseeable epidemics.

The Iranian government closed the international borders to Chinese citizens on 27 February 2020, a seemingly late effort as the COVID-19 cases had already reached 141 and scattered in 20 out of 31 provinces of Iran [3]. On the other hand, a travel ban between cities was implemented on 22 March 2020, while the country was at the summit of the first pandemic wave. Moreover, social and cultural barriers like Iranian new year traditions made the previous attempts futile. Initially, it was believed that the lockdown was a medieval measure. After the importance of lockdown dawned upon the authorities, the economic importance of Iran’s metropolitan cities and the absence of concurrent travel bans exacerbated the dissemination of the disease. Nevertheless, as a critical turning point, the presidential decree for establishing the national COVID-19 committee boosted the inter-sectoral collaboration, provided the appropriate political support to finally overcome the perceived threats of keeping people inside their houses, and facilitated the execution of strategies [4].

Similar to many countries, the rapid pandemic progression outpaced Iran’s health system capacity. More precisely, Iran suffered from a shortage of sufficient Personal Protective Equipment (PPE), ICU beds, available trained medical staff, diagnostic tests, and pharmaceuticals at the very onset of the pandemic. Despite the introduction of effective vaccines, the hesitancy regarding the vaccine import might have contributed to increased casualties. Worse still, the limited vaccine options aggravated the situation, hence the need for unified vaccine recognition patterns among countries [5]. The unfair unilateral sanctions imposed against Iran severely impacted the health sector and hindered the access of Iranians to life-saving gear [6]. Therefore, based on the past and ongoing experience of Iran, to mitigate the risk of future pandemics, we propose: 1) having backup storage of PPE for emergency release; 2) increasing the number of ICU beds with a focus on deprived areas; 3) training health professionals for appropriate crisis management; 4) boosting the cooperation between scientists and decision makers, while defining explicit responsibilities; 5) revisiting the macro-plan for strengthening the required infrastructures to facilitate emergent interventions without the call for aid from the already struggling nations; 6) allocating adequate emergency budget for developing insurance funds and self-isolation paybacks particularly for the poor and vulnerable, who might face more severe consequences of such crisis compared to well-off people [7]; and 7) motivating and engaging with volunteer groups to provide people with necessary supplies during the lockdown.

Iran was ranked third out of 22 in the Eastern Mediterranean Region (EMRO) in establishing an electronic health system [8]. According to the COVID-19 Strategic Preparedness and Response Plan, the Iranian COVID-19 surveillance system showed acceptable performance, including a regular update of demographic and clinical data, actively tracing cases, and illustrating transmission characteristics. Nevertheless, not utilizing its extensive primary healthcare (PHC) network for case finding and community engagement, dysfunctional follow-up for asymptomatic cases in the early pandemic, and initial shortages of diagnostic kits in remote regions hampered disease surveillance [9]. Let alone, limited access to epidemiological data and accessible registries led researchers struggle to conduct proper data analysis and timely monitoring.

Risk communication is one of the prominent pillars of fighting against pandemics [10]. At the beginning of the COVID-19 crisis, information dissemination was slow nationwide, which might have occasionally led to underestimating the potential of virus spread in Iran. The Ministry of Health and Medical Education (MoHME) gradually began to use various media, i.e., daily text messages, national television channels, phone calls, and billboards, to enhance public literacy and restrain infodemic [11]. We advocate fostering proactive, efficient, and timely communication strategies to lower the risk of likely upcoming pandemics. Community engagement is crucial in risk reduction. In this regard, the authorities’ meaningful liaison with reference groups, e.g., religious and cultural leaders is of triumphant importance to disseminate reliable information among the target population.

Future pandemics are likely to happen. Similar to many settings, the Iranian population is aging, with over 65 years old citizens expected to rise from 10.5% in 2025 to 21.7% in 2050 [12]. This might increase Iran’s susceptibility to the deleterious impact of future pandemics. Iran was also ranked third among 17 countries considering the proportion of vaccine hesitancy, which emboldens the necessity of interventions to reshape the community’s attitude (27.9%) [13]. Finally, with people losing their lives and jobs, the insufficient psychosocial support imposed an extra detrimental burden on the population. Indeed, we need universal health coverage, universal health preparedness and universal health solidarity, in line with the whole of government and whole of society approach, to determine how well we will combat the future crisis.
